# The Use of Meta-Analysis for the Measurement of Animal Disease Burden: Losses Due to Clinical Mastitis as an Example

**DOI:** 10.3389/fvets.2020.00149

**Published:** 2020-03-18

**Authors:** Didier Raboisson, Ahmed Ferchiou, Beate Pinior, Thomas Gautier, Pierre Sans, Guillaume Lhermie

**Affiliations:** ^1^IHAP, Université de Toulouse, INRAE, ENVT, Toulouse, France; ^2^Food Technology and Veterinary Public Health, Institute of Food Safety, University of Veterinary Medicine Vienna, Vienna, Austria; ^3^ALISS, INRAE, Ivry-sur-Seine, ENVT, Toulouse, France

**Keywords:** clinical mastitis, economics, etiology, meta-analysis, dairy cows

## Abstract

The literature contains an extensive panel of studies focusing on the costs of animal diseases. The losses of an agriculture holding can be influenced by many factors since farming is a complex system and diseases are closely interrelated. Meta-analysis can be used to detect effects (i.e., change in clinical mastitis losses here) across studies and to identify factors that may influence those effects. This includes the external validity of the published study results with regard to the input parameters and the internal validity of the study, particularly how other diseases related to the target disease were accounted for. Mixed-effect meta-regressions were performed to estimate the mean clinical mastitis losses per case across the literature and to elucidate to what extent clinical mastitis losses are influenced by (i) general factors, such as etiology; (ii) the types of losses that contribute to the total mastitis losses; and (iii) prices. In total, 82 observations from nine studies were included in the meta-analysis to assess mean clinical mastitis losses per case. The multivariate meta-regression showed that etiology significantly influenced the clinical mastitis loss per case. The mean loss was determined to be €224 per case for all published etiologies. In detail, mean losses equalled €457 and €101 per case of clinical mastitis due to gram-negative and gram-positive bacteria, respectively, and €428 and €74 per case of clinical mastitis due to *Escherichia coli* and *Staphylococcus aureus*, respectively. Additionally, the mean loss obtained depended on whether diagnostic costs and reduced feed intake in cases of mastitis were included in the clinical mastitis loss calculation. The monetary values of labor cost, drug cost and culling cost, as well as treatment price (all included), significantly influenced the clinical mastitis losses per case. All other tested moderators were not associated with mastitis losses, highlighting the need for more standardized economic studies, for both methods and ways results are presented, and suggesting that the mastitis losses assessed in the literature cannot be extrapolated (limited external validity). Although meta-analyses are useful to overview the burden of diseases across studies, their ability to summarize extensive literature with various economic assessments is limited. These limitations in loss assessments also suggest the need to focus on management strategies rather than on pure monetary estimations of disease costs, at least for production diseases at the farm level.

## Introduction

Some reviews show large variations in the calculated impact of animal diseases, such as subclinical ketosis bovine viral diarrhea (1–3). There is increasing concerns of cost evaluation in the context of animal diseases because of difficulties in assigning disease cost to an individual disease due to the co-existence of simultaneous diseases (4). Consequently, the risk of overestimating by including the same contributors in the costs of different diseases is high (5–7). Further, key questions in disease impact evaluation is whether and how the results can be extrapolated, particularly considering the high price volatility of input and output parameters in economic assessments. Trends that focus on the whole economic strategy to manage disease rather than on the cost of disease to address this concern are increasing (5, 8). We hypothesize that meta-analysis may be an adequate approach to define how factors such as the type of incorporated losses and the associated prices may influence the value of the economic burden of the disease across the studies.

Mastitis is one of the most important diseases in dairy farms worldwide (9, 10); it is related to economic, environmental and societal stakes through losses, increased carbon and nitrogen outputs from the production process, and increased antimicrobial use (11, 12). The cost of mastitis differs across studies (1, 13, 14), particularly regarding etiology, clinical degree, types of losses included in the mastitis costs, treatment costs, level of prices, and economic assessments methods used. Mastitis is a complex disease, and its diagnosis can be clinical, bacteriologic, and cytologic. Clinical mastitis includes local and general clinical signs, and subclinical mastitis is diagnosed when no clinical signs are observed. A bacteriological diagnosis includes the identification of the etiology of mastitis and antibiotic sensitivity of the pathogen. A cytologic diagnosis is based on milk somatic cell counts (SCCs), which is a proxy generally used to measure subclinical mastitis, despite clinical mastitis lead to high SCCs.

The present work focuses on the factors that may influence losses due to animal diseases, using clinical mastitis as an example, to define whether the present state of the economics of this disease can be adapted to elucidate the (i) internal validity of the study (what is accounted for during the economic assessment) and (ii) the external validity of published study results with regard to the input parameters used. It aims to describe the usefulness of meta-analysis to evaluate which factors may influence the estimated losses due to mastitis infections in the dairy population according to the literature.

## Materials and Methods

### Literature Search and Criteria for the Inclusion or Exclusion of Studies

Publications on the losses due to clinical mastitis were selected from English-language literature up to June 2019. The literature search was conducted in PubMed, Science Direct, and Google Scholar. The keywords were applied separately or in different combinations for the literature search in the three databases. Subsequently, the reference lists of the identified studies were also screened. All the studies were analyzed according to the following inclusion criteria: (i) the publication included clinical mastitis and presented results for clinical mastitis separately from subclinical mastitis if both were included; (ii) the publication included the monetary losses presented per clinical mastitis case or per year and cow; (iii) the publication results were obtained in temperate-climate countries; and (iv) the publication data was obtained after 1990 to represent the modern livestock system. No restrictions were set on the intensification level (milk production), the level of monetary losses due to clinical mastitis, or the currency used. Publications focusing only on the preventive costs of clinical mastitis and/or on specific breeds (Simmental) and/or determinist methods with no variance associated with the mean losses were excluded. In the present work, production losses and curative extra costs were eligible to be considered as clinical mastitis losses. All expressions and proxies of the variance were accepted [i.e., standard deviation (SD), standard error (SE), min-max, confidence intervals] and transformed into a unique unit (i.e., SE) to compare the results across the included studies.

**Figure 1:**
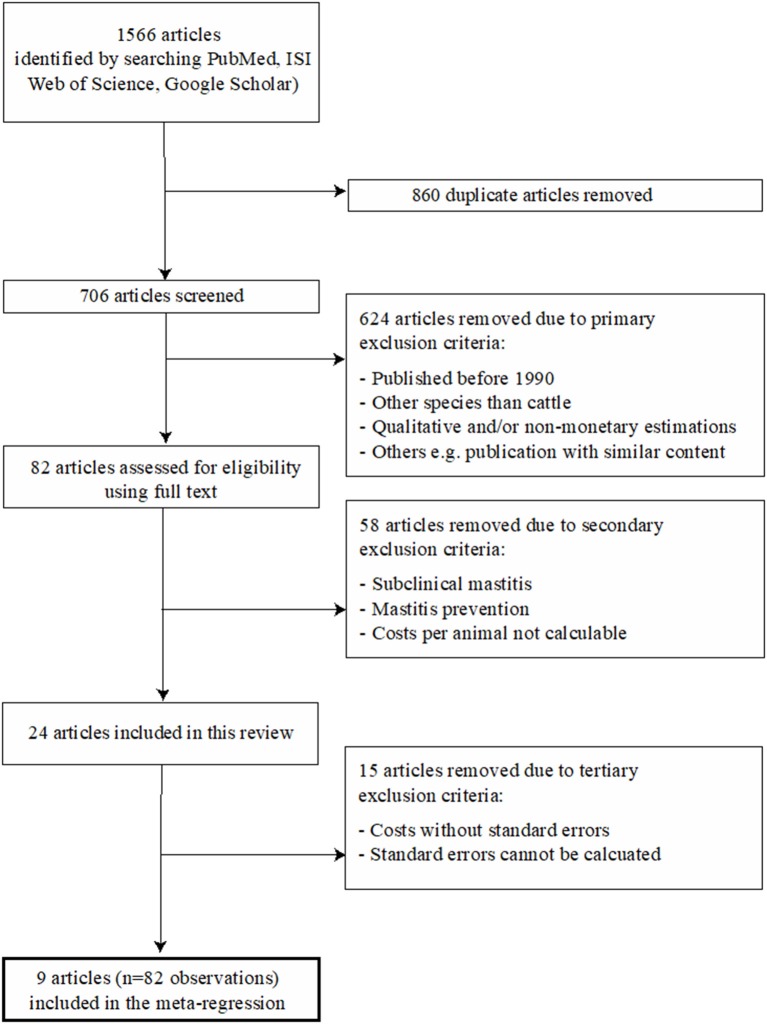
Flow diagram of the selection process for the systematic literature review. The final regressions included eight articles, since the influential analysis has led to exclude one article.

The total number of identified publications and the applied two-step selection process for eligible studies, which was performed in accordance with the PRISMA guidelines (Preferred Reporting Items for Systematic Reviews and Meta-Analysis), are illustrated in Figure 1. All articles were screened in full by two reviewers (TG, DR) and eligible studies, i.e., those which met the inclusion criteria, were then reviewed in full by one reviewer (TG) in accordance with the predefined variables shown in Table 1. All relevant data from the eligible studies were entered into a Microsoft Excel spreadsheet (Table S1). A publication was further divided into different observation sets if the study considered different variables, according to Table 1, into account and thus published different monetary losses per animal. Consequently, the total number of publications included in the presented study was not identical to the total number of observations. The details of the four incorporated groups of variables (called moderators) are reported in Table 1. In brief, they refer to (A) general variables (year, etiology), (B) the type of losses (i.e., nature of the contributors of the losses) considered either included or excluded from the published raw models, (C) the monetary level of losses for each contributor (in Euros), and (D) the prices used as input parameters in the published raw models.

**Table 1:** Factors selected from the systematic review and considered in the meta-regression analysis.

**Moderators**	**Class**	**Number of observations**	**Mean (± SE) value of the clinical mastitis losses (in €)**	**Meta-regression set in which the data is included**
**(A) GENERAL**				
Study type	Modeling	42	293 ± 105	1, 2, and 3
	Descriptive	40	227 ± 159	
Publication year	Numeric	82	262 ± 137	1, 2, and 3
Country	Nominal	82	262 ± 137	1, 2, and 3
Number of herds	Numeric	51	267 ± 145	1
Average herd size	Numeric	49	261 ± 146	1
Number of clinical cases	Numeric	20	291 ± 129	1
Average milk yield^a^	Numeric	35	305 ± 110	1
Parity	All	68	245 ± 144	1
	Primiparous	6	287 ± 19	
	Multiparous	8	379 ± 17	
Incidence (%)	0.12	6	287 ± 19	1
	0.20-0.24	8	379 ± 17	
	0.35	1	87	
Prevalence (%)		48	225 ± 55	1
Etiology 1	All pathogens	48	282 ± 112	1
	Gram positive	16	155 ± 68	
	Gram negative	6	477 ± 119	
	Other (no growth, two pathogens)	12	235 ± 173	
Etiology 2	All	48	282 ± 112	1
	*S. aureus*^e^	4	123 ± 30	
	S. coag.^e^	4	168 ± 104	
	S. spp.^f^	8	165 ± 64	
	Gram negative	6	444 ±108	
	(Other no growth, two pathogens)	12	264 ± 195	
Etiology 3	All	48	282 ± 112	1
	*S. aureus*^d^	4	123 ± 30	
	S. coag.^e^	4	168 ± 104	
	Streptococcus Esculine +	4	152 ± 15	
	Streptococcus Esculine -	4	178 ± 95	
	Gram negative	6	444 ± 108	
	Other (no growth, two pathogens)	12	264 ± 195	
**(B) TYPE OF lOSSES: CONTRIBUTORS TO MASTITIS LOSSES (ACCOUNTED FOR OR NOT)**				
Diagnosis (before treatment)^b^	No	76	261 ± 140	1
	Yes	4	281 ± 15	
Feed intake (saved if mastitis)^c^	No	46	221 ± 151	1
	Yes	36	311 ± 97	
Milk withdrawal	No	0		No
	Yes	82	262 ± 137	
Milk not produced	No	4	87 ± 17	1
	Yes	78	271 ± 134	
Veterinary cost	No	20	291 ± 129	1
	Yes	62	252 ± 139	
Drug cost	No	0		No
	Yes	82	262 ± 137	
Extra labor	No	3	87 ± 21	1
	Yes	77	268 ± 135	
Culling	No	4	87 ± 17	1
	Yes	76	271 ± 134	
Extended day open	No	66	245 ± 144	1
	Yes	14	340 ±50	
Cow Mortality	No	19	175 ±74	1
	Yes	63	289 ±141	
Carcass disposal	No	19	175 ± 74	1
	Yes	63	289 ± 141	
Milk replacer used	No	32	257 ± 125	1
	Yes	48	265 ± 145	
**(C) MONETARY LEVEL OF LOSSES (VALUE OF EACH CONTRIBUTOR)**				
Milk withdrawal		13	261 ± 78	2
Milk not produced		24	245 ± 83	2
Veterinary cost		16	221 ± 100	2
Drug cost		24	328 ± 78	2
Extra labor		26	239 ± 87	2
Culling		25	245 ± 83	2
Extended day open		2	328 ± 61	No
Cow Mortality		3	306 ± 57	No
**(D) PRICES (OF INPUT PARAMETERS OF RAW mODELS)**				
Cow culled (€/kg carcass)	1.69	12	341 ± 50	3
	1.94	2	334 ±70	
Replacement heifer (€/head)	1502	12	341 ± 51	3
	1684	2	330 ± 59	
Milk (€/kg)	0.31	12	375 ± 82	3
	0.33-0.37	15	316 ± 83	
	0.41-0.49	5	366 ±62	
Feed (€/ kg) dry matter	0.16	12	345 ± 48	3
	0.18	2	307 ± 64	
Labor (€/h)	5.89-10.58	13	353 ± 112	3
	19.42-23.88	16	199 ± 64	
	28.88-30.36	14	340 ± 50	
	36.76	35	224 ±153	
Treatment (€/treatment, all included)	Numeric	32	253 ±124	3

a 305-days average milk production;

b mastitis diagnosis before treatment;

c adjustment for reduced feed intake in cases of mastitis;

d Staphylococcus aureus;

e coagulase negative- Staphylococcus;

f *Escherichia coli*.

The clinical mastitis losses reported in the literature were standardized per case of clinical mastitis. The mastitis losses were published in different national currencies and years. A standardization to the Euro (€) and the year 2018 for each respective country was performed as follows:

(1) $$\begin{array}{rcl} {Y\text{ \_}DL}_{\left(\text{€};\;2018\right)} & = & \;\frac{{\;Y\text{ \_}DL}_{\begin{array}{c} X \end{array}}^{i}}{\tau_{{conv^{X}}_{\left(i\to \text{€}\right)}}}*\\ \left(\;\frac{{I\text{ \_}OCDE}_{2018\;}}{{I\text{ \_}OCDE}_{\text{X}}}\right) \end{array}$$

where *Y_DL (€; 2018)* represents the mean clinical mastitis losses per animal in € in 2018, and *i* indicates the national currency of the respective country for which losses were determined in the year *X*. The nominal exchange rate ($\tau_{{{conv\;}^{X}}_{\left(i\to \text{€}\right)}}$) was distinguished between the Eurozone (i.e., exchange rate of the national currency *i* into the currency € in 2002) and non-Eurozone (i.e., exchange rate of the national currency *i* into the currency € in the year of publication). The index I_OCDEx includes the economic annual growth rate of the respective country and incorporates the inflation rate based on the consumer price index. The same procedure of standardization to the Euro (€) and the year 2018 was applied for all monetary values in the dataset (Table S1).

### Meta-Analysis

**Figure 2:**
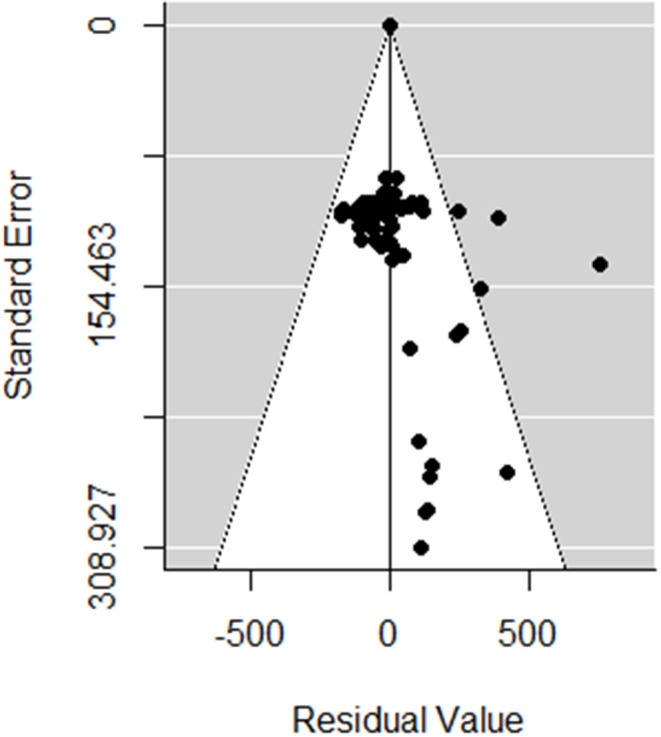
Funnel plot of the random meta-analysis of studies, without the incorporating moderators in Table 1.

The meta-analyses were implemented in R (Version 3.5.1 R Foundation for Statistical Computing, Vienna, Austria) using the Metafor package (15). Random-effects models were first used to estimate the log-effect size and its 95% confidence interval (CI) and statistical significance level. The statistical heterogeneity between and within studies was assessed using the Cochran Q statistic and the *I*^2^ statistic, respectively (16). For response variables with high *I*^2^, uni- or multivariate meta-regression was then performed to explore the sources of heterogeneity. The meta-regression was conducted by screening for the moderators, as described in Table 1. A moderator was a variable that resulted in reduced heterogeneity when introduced in the meta-regression (i.e., factor). In the first step, the meta-regression was performed for all factors together and then separately for (A) the general factors, (B) the type of losses (i.e., contributor), (C) the monetary level of losses for each contributor, and (D) the prices used for the economic input parameters. The variable “Publication” was kept as a random effect. The inclusion of the factors in the meta-regression analysis was conducted as follows. Univariate meta-regressions were first performed to identify factors according to Table 1 that may have had a significant association with the clinical mastitis loss per case. A reference class for each factor was chosen to allow a comparison of the effect size. Any significant factors in the univariate test were selected as a potential influencing factor for the multivariate analysis, which aimed to reduce the heterogeneity between the included studies in the meta-analysis. The τ^2^ (residual heterogeneity variance) denoted the amount of heterogeneity that may not be explained through the inclusion of the factors in the meta-analysis. Publication bias was identified by performing the Egger test, a regression test for funnel plot asymmetry and inspection of the associated funnel plots (Figure 2). Outliers were also identified by conducting influential case diagnostics (i.e., DFFITS value, Cook's distances, covariance ratios, estimates of τ^2^ and test statistics for (residual) heterogeneity). Because the dataset contained moderators with different numbers of missing data, three subdatasets were proposed, as described in Table 1, and meta-regression was performed accordingly.

## Results

In total, 82 observations from nine studies were included in the meta-analysis (Table S2). Influential case diagnostics indicated two observations and one study as sources of asymmetry. These observations were considerably higher regarding clinical mastitis loss per case (with a mean of €1,000) than other observations (with a mean ± SD of €262 ± 137) and thus highly influenced the results of the meta-regression. Consequently, two observations and one study were excluded in the presented meta-analysis. The estimated pooled effect size obtained with the random-effects model with no moderator (Table 2) was €195 (Se = 37, *p* < 0.001). No publication bias was determined with the Egger test (*t* = 269, *p* = 0.21). The heterogeneity between the studies was very high (*I*^2^ = 99.9%; AIC = 99,711; Q-Test: *x*^2^ = 99,136; df = 79; *p* < 0.001).

**Table 2:** Final factors considered in the meta-regression analysis.

**Group of moderators^a^**	**Moderator and class**	**Estimate (SE)**	**95% CI**	***P*-value**
Without	Intercept	195 (37)	122/267	<0.0001
A and B: General and type of mastitis losses	Intercept	224 (43)	139/308	<0.0001
	Gram positive (ref=All)	−123 (7.3)	−108/−137	<0.0001
	Gram negative (ref=All)	233 (16.3)	201/264	<0.0001
	Other (ref=All)	−133 (7.0)	−119/−146148/161	<0.0001
	Diagnosis (ref= No)	155 (3.5)	148/161	<0.0001
	Feed intake (ref = No)	−29 (2.5)	−24/−34 139/308	<0.0001
A and B: General and type of mastitis losses ^b^	Intercept	224 (43)	139/308	<0.0001
	*Staphylococcus aureus* (ref=All)	−150 (9)	−132/−167	<0.0001
	*Staphylococcus* spp. (ref=All)	−145 (11)	123/166 166166	<0.0001
	*Streptococcus* spp. (ref=All)	−103 (8)	−87/−118	<0.0001
	*E. coli* (ref=All)	204 (18)	168/239	0.0097
	Other (ref=All)	−131 (7.0)	−117/−144	<0.0001
	Diagnosis (ref= No)	155 (3.5)	148/161	<0.0001
	Feed intake (ref = No)	−29 (2.5)	−24/−34	<0.0001
C– Monetary level of losses^c^	Intercept	124 (43)	39/208	0.0039
	Labour^e^	2.9 (0.24)	2.4/3.4	<0.0001
	Drug cost^e^	0.8 (0.04)	0.72/0.87	<0.0001
	Culling^e^	1.04 (0.02)	1.00/1.08	<0.0001
D- Prices^d^	Intercept	150 (39)	73/226	<0.0001
	Treatment (all included)^f^	0.76 (0.04)	0.68/0.83	<0.0001

a *as defined in Table 1*.

b, c, d *the corresponding influential case diagnostics are indicated in Figures S1, S2, and S3, respectively*.

e *the moderator is a continuous variable that equals the monetary value of the contributor of the losses due to clinical mastitis. The coefficient is then expressed as the marginal value of loss (for one extra euro of the total value of the contributors “labor cost,” “treatment cost,” and “culling cost”)*.

f *the moderator is a continuous variable that equals the price of treatment considered in the raw model. The coefficient is then expressed as the marginal value of loss (for one extra euro of treatment price)*.

The meta-regression with all moderators, which was performed on the dataset including the moderators without any missing data (denoted subdataset 1 in Table 1), showed that mastitis losses were associated with the etiology of mastitis as well as with the inclusion diagnostic costs and feed intake decreases in cases of mastitis in the raw model (Table 2). The observed decrease in the heterogeneity through the inclusion of these factors was 33%. A diagnosis before treatment was associated with an extra loss of €155 per case, and the adjustment of the cost assessment by the diet saved with a reduced the loss per case by €35. The average losses of gram positive and gram negative clinical mastitis were €101 (€224–€123) and €457 (€224+€233), respectively. The losses were €74 (€224–€150), €79 (€224–€145), €121 (€224–€103) and €428 (€224+€204) for mastitis due to *Staphylococcus aureus*, coagulase-*Staphylococcus, Streptococcus* spp., and *Escherichia coli*, respectively (Table 2).

The dataset focusing on the monetary levels of losses includes 26 observations (denoted subdataset 2, see Table 1). All moderators summarized in the term monetary levels of losses in Table 1 were significantly associated with the cost of mastitis, but correlation was observed between the moderators (Table 3), leading to the final regression proposed in Table 2. The marginal values of drug cost, labor cost and culling cost in clinical mastitis losses were €0.8, €2.9, and €1.04, respectively. This means, for instance, that one extra euro for the drug cost was associated with an extra clinical mastitis losses equal to €0.8.

**Table 3:** Correlations (and *P-*values) of the values of the moderators in the category “monetary level of losses” (moderator group C, see Table 1).

	**Milk not produced**	**Milk withdrawal**	**Veterinary cost**	**Drug cost**	**Extra labor**	**Culling**
Milk withdrawal	0.92/<0.001^a^					
Veterinary cost	0.92/<0.001	0.94/<0.001				
Drug cost	−0.02/0.89	0.36/0.06	0.43/0.06			
Extra labor	0.77/<0.001	0.9/<0.001	0.85/<0.001	0.04/0.8		
Culling	0.68/<0.001	0.6/<0.001	0.53/0.005	−0.34/0.9	0.39/0.01	
Mortality	−0.2/0.38	0.32/0.26	−0.61/<0.001	0.61/0.01	0.67/0.09	0.78/0.06

a *Expressed as correlation value/P-value*.

The dataset focusing on prices of inputs included 12 to 76 observations, depending on the moderators (denoted subdataset 3, see Table 1). The price of treatment (all included) was associated with a marginal value in the clinical mastitis-related loss of €0.76 (Se = 0.04, *p* < 0.0001). All other moderators, including the price of milk, were not associated with the losses of clinical mastitis (*p* > 0.7 for the three classes compared to the reference class, Table 1).

**Figure 3:**
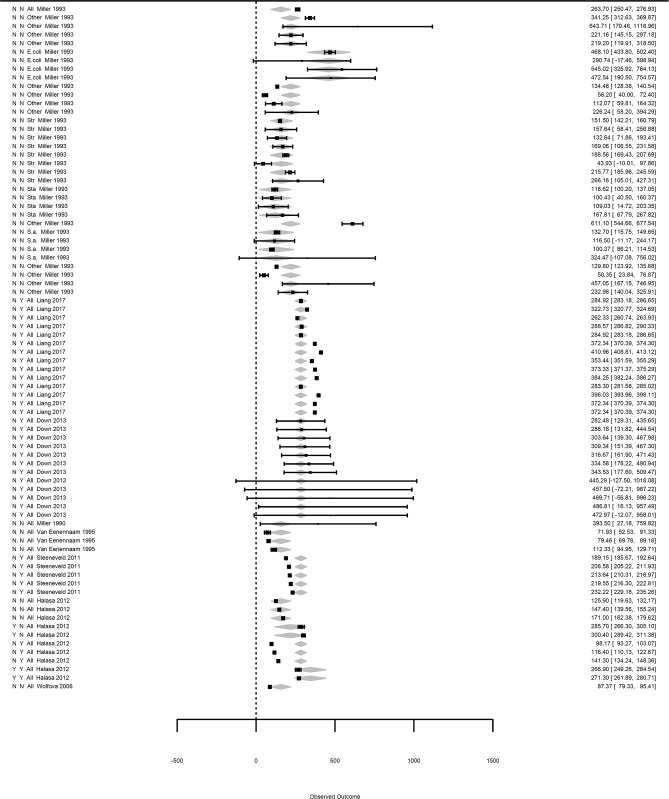
Forest graph of the meta-regressions including moderators in groups A and B. The column on the right refers to the mean loss per case with the corresponding confidence interval (in brackets). The two single letters in the left column represent the moderators diagnosis and feed intake, as defined in Table 1. The moderator etiology (Table 1) is located to the left of the authors. The gray diamonds represent the effect size adjusted for the moderator and are included in the meta-regression.

**Figure 4:**
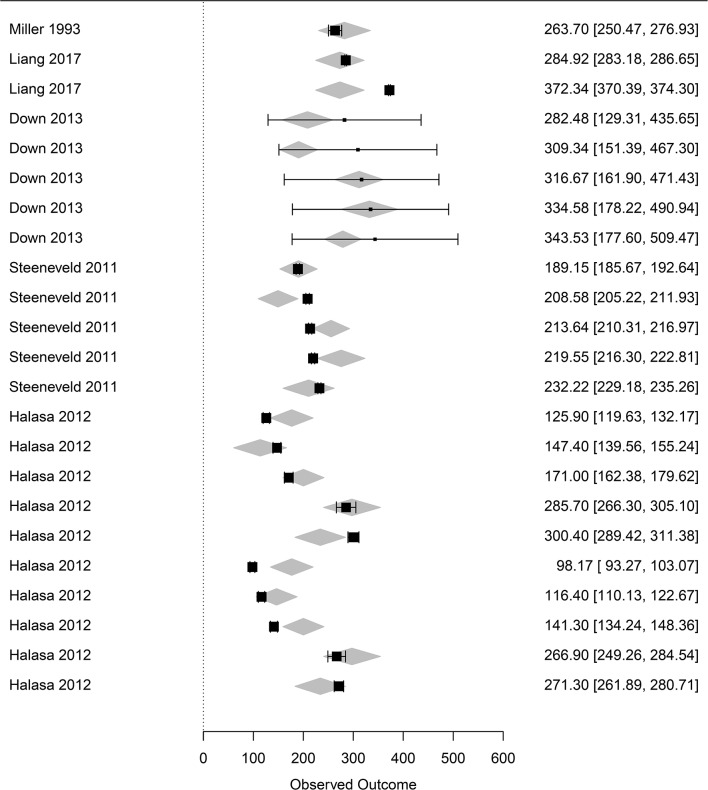
Forest graph of the meta-regression including moderators in group C (see Table 1). The column on the right refers to the mean loss per case with the corresponding confidence interval (in brackets). The gray diamonds represent the effect size adjusted for the moderators included in the meta-regression.

**Figure 5:**
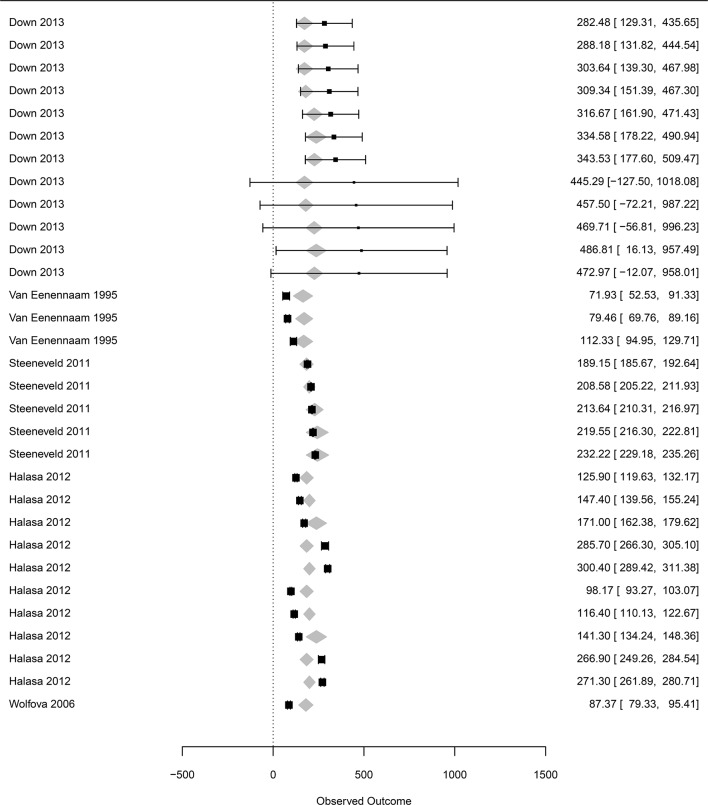
Forest graph of the meta-regression including moderators in group D (see Table 1). The column on the right refers to the mean loss per case with the corresponding confidence interval (in brackets). The gray diamonds represent the effect size adjusted for the moderator included in the meta-regression.

The influential case diagnostics of the three meta-regressions shown in Table 2 indicated outliers in the incorporated influencing factors on mastitis losses (Figures S1–S3). The removal of the outliers did not change the coefficients of the meta-regressions, and the results shown in Table 2 were considered final. Final forest plots are reported in Figures 3–5.

## Discussion

The meta-regression was performed according to usual recommendations (17, 18). The final choice for the models was made considering the decrease in heterogeneity. More than one model was reported for the same outcome because the authors judged that all the models had biological significance and would be of interest to the scientific community. The multivariate models provided in Table 2 show close coefficients compared to the univariate models, and the addition of a new moderator reduced the heterogeneity. Unfortunately, many studies did not report any estimation of the variance (determinist method) and could not be included in the present meta-regression, leading to only nine included publications, although extensive literature is available. This issue has been highlighted in a previous review (13). The present meta-analysis and the previous review (13) both highlight the high heterogeneity within the method used to assess mastitis losses, the nature of the included losses and the limits of comparing or summarizing results. In spite that one aim of the present work was to adjust the estimation of clinical mastitis losses by the occurrence of other diseases, data available did not permit to do for most of the regressions since papers included in the meta-analysis only scarcely reported other diseases that may interact with mastitis. Moreover, even if many non-significant associations were found in the present study, it helps (i) to determine factors influencing mastitis losses, (ii) to quantify heterogeneity, and (iii) to highlight the concerns faced when aiming at reducing heterogeneity in the context of clinical mastitis losses.

The present study should remind the reader that focusing on the cost or loss of disease may be of limited value for some diseases, such as mastitis. The present results show that the losses of clinical mastitis were higher for gram negative mastitis than for gram positive mastitis, although opposite perception has been reported from the field. For instance, the management of gram negative mastitis is easier than gram positive mastitis, although the long-term consequences of udder contamination by gram positive are greater than those for gram negative mastitis (treatment efficacy, chronic infection). This result is in accordance with the fact that medium- or long-term SCCs arising from clinical mastitis are poorly accounted for in the present works since they are scarcely reported in the literature. Similarly, the way culling is integrated in the studies remains unclear. This result demonstrates that any economic reasoning focusing on the losses of mastitis is inappropriate and should be avoided. The present meta-analysis does not provide additional information since the economic reasoning in most of the raw models used in the present meta-analysis was biased. Recent literature on economics of udder health has increasingly focused on the strategic management of mastitis instead of its losses (19–21). This appears to be an appropriate trend since it accounts for herd dynamics, short- and long-term issues and farmers' behaviors, for instance, through farmers' satisfaction not only relying on income optimization (utility). This is also a reason why some recent publications cannot be included in the present meta-analysis that focus on clinical mastitis losses. The exclusion criteria have also led to focus on the period from 1990 to 2019. The milk production environment has changed a lot worldwide during this period. The period was included in the present analysis as moderator but it was not significantly associated with the clinical mastitis losses. Altogether, the PRISMA procedure, the outcome studied, the period focused and the geographical restrictions have led to exclude many studies that deal with economics of mastitis.

The present work tried to explain the losses of clinical mastitis considering different groups of moderators (Table 1). The moderator groups B, C, and D (see Table 1) referred to the questions “is the moderator accounted for? (yes/no),” “can the size of the contributor (total monetary value) be linked to clinical mastitis losses?” i.e., “is the share of the contributor almost always the same?” and “can we summarize how the economic input parameters influence the total losses?,” respectively. Moderator group B showed that factors were systematically included, scarcely included (adjustment for reduced feed intake), or almost never included (reproduction impact, pre-treatment diagnosis). These differences contributed to the large heterogeneity observed in the outcome of the present study. Unfortunately, moderators in group C were scarcely reported. Publications precisely reported the main contributors included in the economic assessment (moderators in group B), but most of them failed to clearly describe the value of this contributor (moderators in group C). This is a key limit to evaluating whether the share of the contributors substantially changed between publications, and it does not allow here to draw a conclusion on the external validity of the literature, even if it is extensive. Such a standardization would require precise lists of items to be reported and clear procedures to be followed to perform and evaluate the analysis, as it exists for other scientific approaches (see PRISMA as the reference method for meta-analyses). In a companion paper focusing on BVD, the same limits were highlighted, and the contributors of the total losses of BVDV infection could not be defined precisely due to imprecision in the included contributors and a lack of clear reporting of the values of each contributor (4). Last, the values of the economic parameters had almost no association with the clinical mastitis losses. This is also due to unclear results in the publications and the low variability within the values of the moderators. In addition, no association was reported between the input values and the outcome for moderators such as price of milk or labor (Table 1), although the number of trials included was moderate to high and the range of the values appeared to be appropriate for regression. The wide range in the values of milk price without any significant association was in opposition with the statement that the price of milk influences the losses of the disease and may be an item to consider to adapt the disease management strategy to the market context, which is often reported in sensitivity analyses in publications and in the field. The present results do not support such a relationship. Based on the present results and due to the heterogeneity, the conclusion is that the scientific community should be very careful to use the monetary values from one study out of its context or for their own study design, because the mastitis losses depend on many other factors which cause the heterogeneity that could not be explained by the factors considered in the present study.

## Conclusions

The present work proposes an estimation of the losses of clinical mastitis for different etiologies. It failed to elucidate which contributor mainly influenced the losses of clinical mastitis and did not highlight any relationship between the price of milk or labor and losses due to clinical mastitis. This supports the avoidance any economic reasoning focused on the losses of each case of clinical mastitis since such a reasoning is inappropriate from an economic point of view. The internal and external validity of the losses evaluation is highly questionable in the case of clinical mastitis. These results also highlight the need for standardization on how economic assessments of losses due to animal diseases should be performed. This includes both methods and ways results are presented.

## Data Availability Statement

All datasets generated for this study are included in the article/Supplementary Material.

## Author Contributions

DR and TG designed and performed the analysis. BP, AF, PS, and GL contributed to the analysis, reviewed, and revised the manuscript.

### Conflict of Interest

The authors declare that the research was conducted in the absence of any commercial or financial relationships that could be construed as a potential conflict of interest.

## References

[1] Raboisson D Mounié M Maigné E. Diseases, reproductive performance, and changes in milk production associated with subclinical ketosis in dairy cows: a meta-analysis and review. J Dairy Sci. (2014) 97:7547–63. 10.3168/jds.2014-8237 25306269

[2] Pinior B Firth CL Richter V Lebl K Trauffler M Dzieciol M. A systematic review of financial and economic assessments of bovine viral diarrhea virus (BVDV) prevention and mitigation activities worldwide. Prev Vet Med. (2017) 137:77–92. 10.1016/j.prevetmed.2016.12.014 28040270

[3] Richter V Lebl K Baumgartner W Obritzhauser W Käsbohrer A Pinior B. A systematic worldwide review of the direct monetary losses in cattle due to bovine viral diarrhoea virus infection. Vet J. (2017) 220:80–7. 10.1016/j.tvjl.2017.01.005 28190502

[4] Pinior B Garcia S Minviel JJ Raboisson D. Epidemiological factors and mitigation measures influencing production losses in cattle due to bovine viral diarrhoea virus infection: a meta-analysis. Transbound Emerg Dis. (2019) 66:2426–39. 10.1111/tbed.13300 31328411 PMC6900039

[5] Raboisson D Ferchiou A Lhermie G Sans P Multi-criteria Optimisation to Fix the Limits of Present Standards in Microeconomics of Animal Health: The Example of Dairy Production. Atlanta, GA: The International Society for Economics and Social Sciences of Animal Health (Atlanta: ISESSAH).

[6] GBADs GBADS – Global Burden of Animal Diseases. (2019). Available online at: https://animalhealthmetrics.org/about/ (accessed November 27, 2019).

[7] Ferchiou A Raboisson D Minviel JJ Bio-economic modelling of antimicrobial use and health management in French dairy production. In: The Agricultural Economics Society Annual Meeting (AES). Coventry (2018). 10.22004/ag.econ.273484

[8] Marschik T Obritzhauser W Wagner P Richter V Mayerhofer M Egger-Danner C. A cost-benefit analysis and the potential trade effects of the bovine viral diarrhoea eradication programme in Styria, Austria. Vet J. (2018) 231:19–29. 10.1016/j.tvjl.2017.11.010 29429483

[9] Hillerton JE Berry EA. Treating mastitis in the cow–a tradition or an archaism. J Appl Microbiol. (2005) 98:1250–5. 10.1111/j.1365-2672.2005.02649.x 15916638

[10] Seegers H Fourichon C Beaudeau F. Production effects related to mastitis and mastitis economics in dairy cattle herds. Vet Res. (2003) 34:475–91. 10.1051/vetres:2003027 14556691

[11] Schabauer A Pinior B Gruber C-M Firth CL Käsbohrer A Wagner M. The relationship between clinical signs and microbiological species, spa type, and antimicrobial resistance in bovine mastitis cases in Austria. Vet microbiol. (2018) 227:52–60. 10.1016/j.vetmic.2018.10.024 30473352

[12] Firth CL Käsbohrer A Egger-Danner C Fuchs K Pinior B Roch F-F. Comparison of defined course doses (DCDvet) for blanket and selective antimicrobial dry cow therapy on conventional and organic farms. Animals. (2019) 9:707. 10.3390/ani9100707 31547125 PMC6826441

[13] Halasa T Huijps K Østerås O Hogeveen H. Economic effects of bovine mastitis and mastitis management: a review. Vet Q. (2007) 29:18–31. 10.1080/01652176.2007.9695224 17471788

[14] Hogeveen H Steeneveld W Wolf CA Production diseases reduce the efficiency of dairy production: a review of the results, methods, and approaches regarding the economics of mastitis. Annu Rev Resour Econ. (2019) 11:289–312. 10.1146/annurev-resource-100518-093954

[15] Viechtbauer W Conducting meta-analyses in R with the metafor package. J Stat Softw. (2010) 36:1–48. 10.18637/jss.v036.i03

[16] Higgins JPT Thompson SG. Quantifying heterogeneity in a meta-analysis. Stat Med. (2002) 21:1539–58. 10.1002/sim.1186 12111919

[17] Schwarzer G Carpenter JR Rücker G. Meta-analysis with R. Cham; Freiburg; London: Springer (2015) 10.1007/978-3-319-21416-0

[18] Endo R Ishii A Nakanishi A Nabenishi H Ashizawa K Tsuzuki Y Effect of the addition of β-hydroxybutyrate to chemically defined maturation medium on the nuclear maturation, sperm penetration and embryonic development of porcine oocytes *in vitro*. Asian-Australas J Anim Sci. (2010) 23:1421–6. 10.5713/ajas.2010.10073

[19] Gussmann M Steeneveld W Kirkeby C Hogeveen H Nielen M Farre M. Economic and epidemiological impact of different intervention strategies for clinical contagious mastitis. J Dairy Sci. (2019) 102:1483–93. 10.3168/jds.2018-14939 30580951

[20] Gussmann M Denwood M Kirkeby C Farre M Halasa T. Associations between udder health and culling in dairy cows. Prev Vet Med. (2019) 171:104751. 10.1016/j.prevetmed.2019.104751 31487555

[21] Getaneh AM Mekonnen SA Hogeveen H. Stochastic bio—economic modeling of mastitis in Ethiopian dairy farms. Prev Vet Med. (2017) 138:94–103. 10.1016/j.prevetmed.2017.01.014 28237241

