# Supplementary material for: The Use of Meta-Analysis for the Measurement of Animal Disease Burden: Losses Due to Clinical Mastitis as an Example
Source: Front Vet Sci. 2020 Mar 18;7:149. doi: 10.3389/fvets.2020.00149 (PMC7093557; doi:10.3389/fvets.2020.00149)
Supplement: Supplementary file 1 [file Data_Sheet_1.PDF]

Supplementary Material

Fig. S1: Influential case diagnostics and the identified outliers (shown as red circles) in the meta-regression including the groups of moderators A and B (see Table 2)

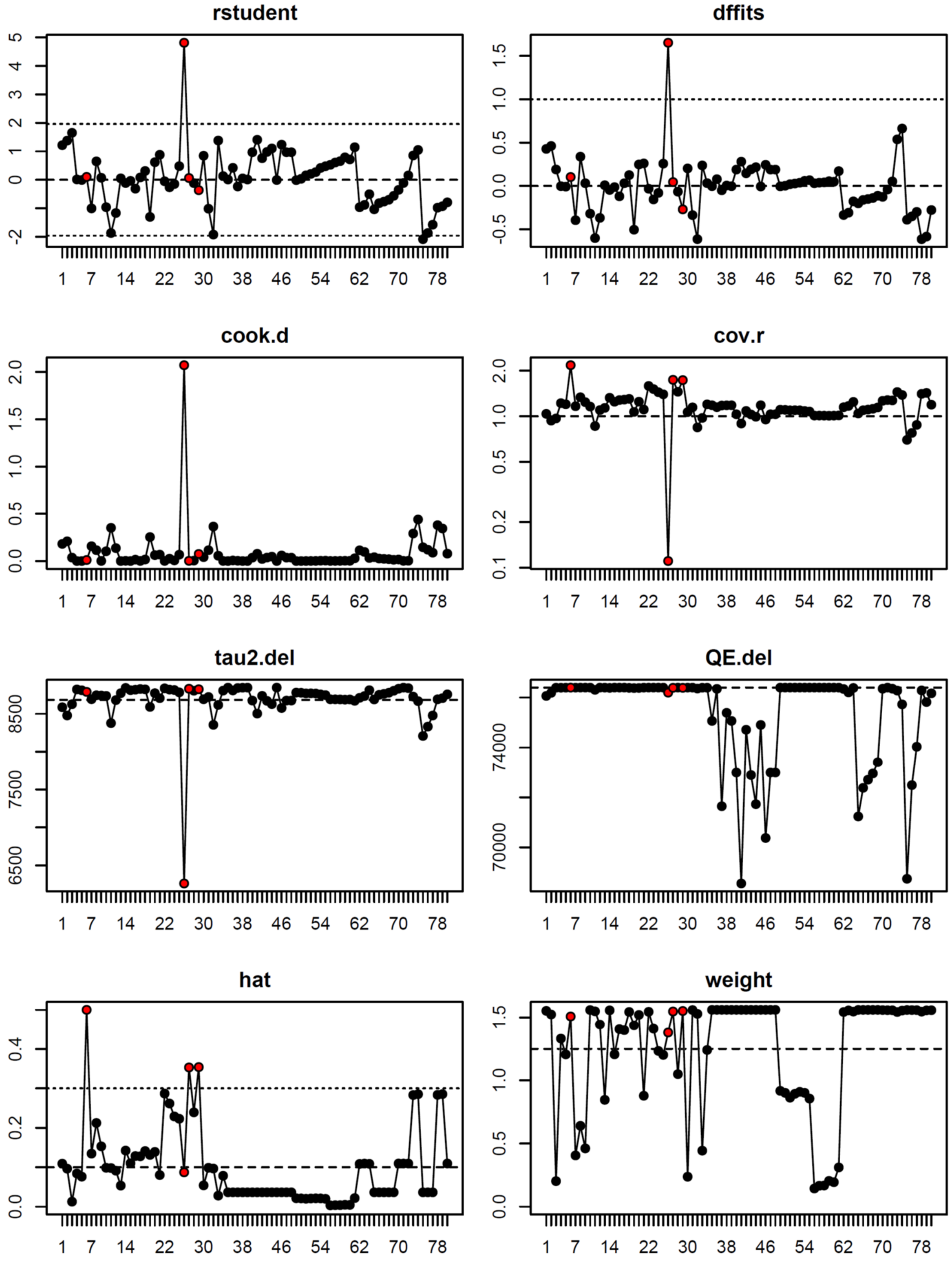

Fig. S2: Influential case diagnostics and the identified outliers (shown as red circles) in the meta-regression including the group of moderator C (see Table 2)

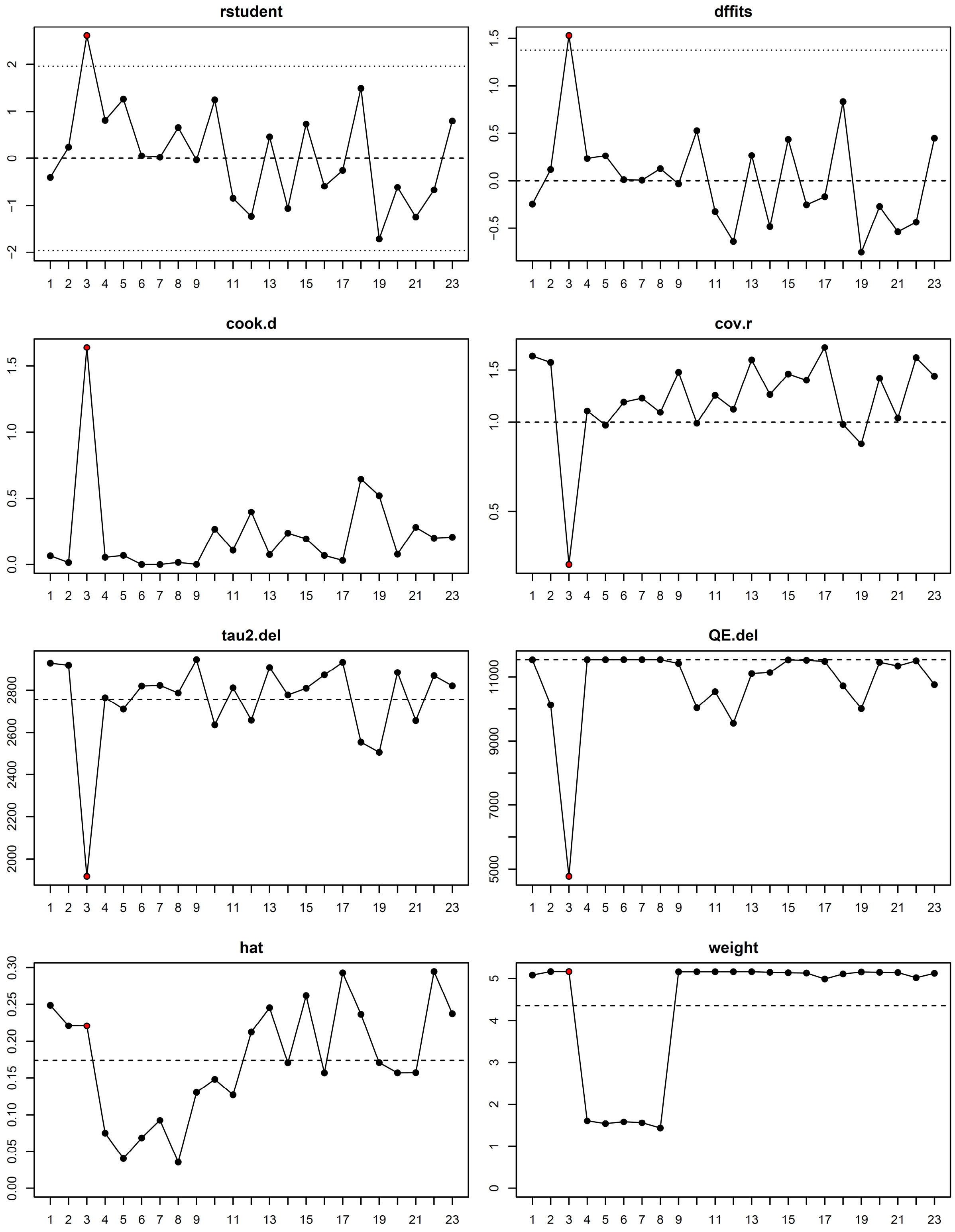

Fig. S3: Influential case diagnostic and the identified outliers (shown as red circles) in the meta-regression including the group of moderator D (see Table 2)

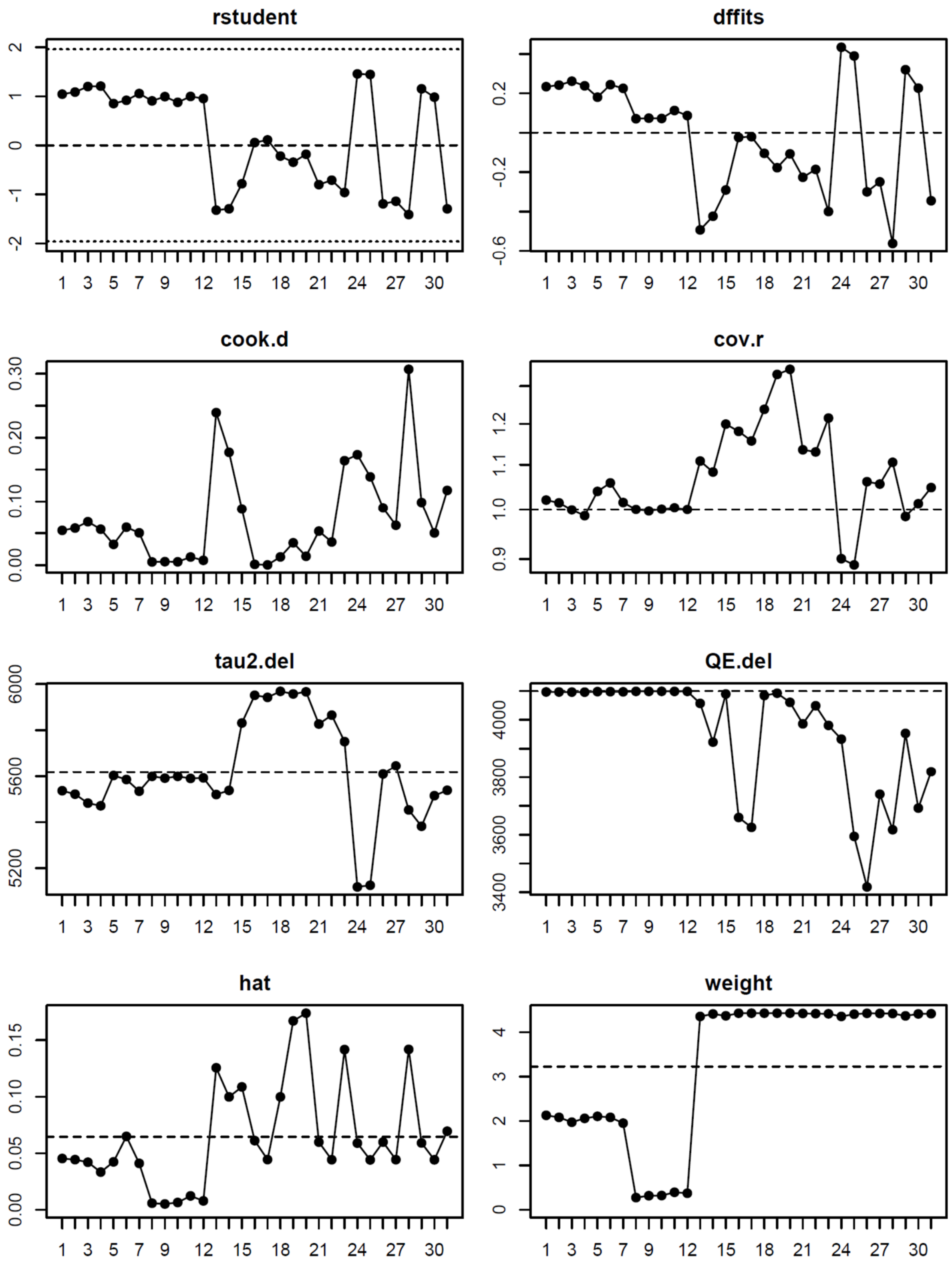

Table S1: Data used for the meta-analysis.

A General factors and aetiology															B-Types of losses : contributors to the mastitis losses (accounted for or not)									
Authors	Year	Study type (Modelling/Descriptive)	Country	Nb Clinical cases	Herdsize	Nb of herds	Parity	Incidence (%)	Prevalence (%)	Aetiology # 1	Aetiology # 2	Aetiology # 3	Cost_2018 (€)(case)	Se_Cost_2018 (€)(case)	Diagnosis (before treatment)	Feed intake (saved if mastitis)	Milk not produced	Veterinary cost	Extra labour	Culling	Extended day open	Cow Mortality	Carcass disposal	Milk replacer used
Miller 1993(1)	1993	D	USA		81	50	All	24%	44.0%	10	10	10	263.70	6.75	0	0	1	1	1	1	0	1	1	1
Miller 1993(1)	1993	D	USA		81	40	All		4.8%	11	11	11	341.25	14.60	0	0	1	1	1	1	0	1	1	1
Miller 1993(1)	1993	D	USA		30	16	All		4.8%	11	11	11	643.71	241.46	0	0	1	1	1	1	0	1	1	1
Miller 1993(1)	1993	D	USA		75	12	All		4.8%	11	11	11	221.16	38.78	0	0	1	1	1	1	0	1	1	1
Miller 1993(1)	1993	D	USA		156	12	All		4.8%	11	11	11	219.20	50.66	0	0	1	1	1	1	0	1	1	1
Miller 1993(1)	1993	D	USA		81	26	All		2.1%	2	5	5	468.10	17.50	0	0	1	1	1	1	0	1	1	1
Miller 1993(1)	1993	D	USA		30	8	All		2.1%	2	5	5	290.74	157.25	0	0	1	1	1	1	0	1	1	1
Miller 1993(1)	1993	D	USA		75	7	All		2.1%	2	5	5	545.02	111.79	0	0	1	1	1	1	0	1	1	1
Miller 1993(1)	1993	D	USA		122	11	All		2.1%	2	5	5	472.54	143.90	0	0	1	1	1	1	0	1	1	1
Miller 1993(1)	1993	D	USA		81	26	All		3.0%	8	8	8	134.46	3.10	0	0	1	1	1	1	0	1	1	1
Miller 1993(1)	1993	D	USA		30	7	All		3.0%	8	8	8	56.20	8.26	0	0	1	1	1	1	0	1	1	1
Miller 1993(1)	1993	D	USA		75	9	All		3.0%	8	8	8	112.07	26.66	0	0	1	1	1	1	0	1	1	1
Miller 1993(1)	1993	D	USA		122	10	All		3.0%	8	8	8	226.24	85.74	0	0	1	1	1	1	0	1	1	1
Miller 1993(1)	1993	D	USA		81	30	All		1.9%	1	3	3	151.50	4.74	0	0	1	1	1	1	0	1	1	1
Miller 1993(1)	1993	D	USA		30	8	All		1.9%	1	3	3	157.64	50.63	0	0	1	1	1	1	0	1	1	1
Miller 1993(1)	1993	D	USA		75	10	All		1.9%	1	3	3	132.64	31.01	0	0	1	1	1	1	0	1	1	1
Miller 1993(1)	1993	D	USA		120	12	All		1.9%	1	3	3	169.06	31.90	0	0	1	1	1	1	0	1	1	1
Miller 1993(1)	1993	D	USA		81	22	All		1.3%	1	3	4	188.56	9.76	0	0	1	1	1	1	0	1	1	1
Miller 1993(1)	1993	D	USA		30	6	All		1.3%	1	3	4	43.93	27.52	0	0	1	1	1	1	0	1	1	1

Miller 1993(1)	1993	D	USA		75	6	All		1.3%	1	3	4	215.77	15.21	0	0	1	1	1	1	0	1	1	1
Miller 1993(1)	1993	D	USA		115	10	All		1.3%	1	3	4	266.16	82.22	0	0	1	1	1	1	0	1	1	1
Miller 1993(1)	1993	D	USA		81	50	All		0.3%	2	6	6	974.00	90.70	0	0	1	1	1	1	0	1	1	1
Miller 1993(1)	1993	D	USA		81	21	All		1.6%	1	2	2	118.63	9.40	0	0	1	1	1	1	0	1	1	1
Miller 1993(1)	1993	D	USA		30	5	All		1.6%	1	2	2	100.43	30.58	0	0	1	1	1	1	0	1	1	1
Miller 1993(1)	1993	D	USA		75	10	All		1.6%	1	2	2	109.03	48.12	0	0	1	1	1	1	0	1	1	1
Miller 1993(1)	1993	D	USA		133	6	All		1.6%	1	2	2	167.81	51.03	0	0	1	1	1	1	0	1	1	1
Miller 1993(1)	1993	D	USA		81	50	All		0.4%	2	9	7	611.10	33.90	0	0	1	1	1	1	0	1	1	1
Miller 1993(1)	1993	D	USA		81	15	All		1.0%	1	1	1	132.70	8.65	0	0	1	1	1	1	0	1	1	1
Miller 1993(1)	1993	D	USA		30	5	All		1.0%	1	1	1	116.50	65.14	0	0	1	1	1	1	0	1	1	1
Miller 1993(1)	1993	D	USA		75	2	All		1.0%	1	1	1	100.37	7.22	0	0	1	1	1	1	0	1	1	1
Miller 1993(1)	1993	D	USA		114	8	All		1.0%	1	1	1	324.47	220.18	0	0	1	1	1	1	0	1	1	1
Miller 1993(1)	1993	D	USA		81	29	All		2.0%	9	9	9	129.80	3.00	0	0	1	1	1	1	0	1	1	1
Miller 1993(1)	1993	D	USA		30	8	All		2.0%	9	9	9	50.35	13.53	0	0	1	1	1	1	0	1	1	1
Miller 1993(1)	1993	D	USA		75	10	All		2.0%	9	9	9	457.05	147.91	0	0	1	1	1	1	0	1	1	1
Miller 1993(1)	1993	D	USA		124	11	All		2.0%	9	9	9	232.98	47.42	0	0	1	1	1	1	0	1	1	1
Liang 2017(2)	2017	M	USA		170.2	10,524	P	12.14%		10	10	10	284.92	0.88	0	1	1	1	1	1	1	1	1	1
Liang 2017(2)	2017	M	USA		170.2	10,524	P	12.14%		10	10	10	322.73	1.00	0	1	1	1	1	1	1	1	1	1
Liang 2017(2)	2017	M	USA		170.2	10,524	P	12.14%		10	10	10	262.33	0.81	0	1	1	1	1	1	1	1	1	1
Liang 2017(2)	2017	M	USA		170.2	10,524	P	12.14%		10	10	10	288.57	0.89	0	1	1	1	1	1	1	1	1	1
Liang 2017(2)	2017	M	USA		170.2	10,524	P	12.14%		10	10	10	284.92	0.88	0	1	1	1	1	1	1	1	1	1
Liang 2017(2)	2017	M	USA		170.2	10,524	M	20.39%		10	10	10	372.34	1.00	0	1	1	1	1	1	1	1	1	1
Liang 2017(2)	2017	M	USA		170.2	10,524	M	20.39%		10	10	10	410.96	1.10	0	1	1	1	1	1	1	1	1	1
Liang 2017(2)	2017	M	USA		170.2	10,524	M	20.39%		10	10	10	353.44	0.95	0	1	1	1	1	1	1	1	1	1
Liang 2017(2)	2017	M	USA		170.2	10,524	M	20.39%		10	10	10	373.33	1.00	0	1	1	1	1	1	1	1	1	1
Liang 2017(2)	2017	M	USA		170.2	10,524	M	20.39%		10	10	10	384.25	1.03	0	1	1	1	1	1	1	1	1	1
Liang 2017(2)	2017	M	USA		170.2	10,524	P	12.14%		10	10	10	283.30	0.88	0	1	1	1	1	1	1	1	1	1
Liang 2017(2)	2017	M	USA		170.2	10,524	M	20.39%		10	10	10	396.03	1.06	0	1	1	1	1	1	1	1	1	1
Liang 2017(2)	2017	M	USA		170.2	10,524	M	20.39%		10	10	10	372.34	1.00	0	1	1	1	1	1	1	1	1	1
Liang 2017(2)	2017	M	USA		170.2	10,524	M	20.39%		10	10	10	372.34	1.00	0	1	1	1	1	1	1	1	1	1
Down 2013(3)	2013	M	UK	4,000			All			10	10	10	282.48	78.15	0	1	1	0	1	1	0	1	1	0
Down 2013(3)	2013	M	UK	4,000			All			10	10	10	288.18	79.78	0	1	1	0	1	1	0	1	1	0

Down 2013(3)	2013	M	UK	4,000			All			10	10	10	303.64	83.85	0	1	1	0	1	1	0	1	1	0
Down 2013(3)	2013	M	UK	4,000			All			10	10	10	309.34	80.59	0	1	1	0	1	1	0	1	1	0
Down 2013(3)	2013	M	UK	4,000			All			10	10	10	316.67	78.96	0	1	1	0	1	1	0	1	1	0
Down 2013(3)	2013	M	UK	4,000			All			10	10	10	334.58	79.78	0	1	1	0	1	1	0	1	1	0
Down 2013(3)	2013	M	UK	4,000			All			10	10	10	343.53	84.66	0	1	1	0	1	1	0	1	1	0
Down 2013(3)	2013	M	UK	4,000			All			10	10	10	445.29	292.25	0	1	1	0	1	1	0	1	1	0
Down 2013(3)	2013	M	UK	4,000			All			10	10	10	457.50	270.27	0	1	1	0	1	1	0	1	1	0
Down 2013(3)	2013	M	UK	4,000			All			10	10	10	469.71	268.64	0	1	1	0	1	1	0	1	1	0
Down 2013(3)	2013	M	UK	4,000			All			10	10	10	486.81	240.15	0	1	1	0	1	1	0	1	1	0
Down 2013(3)	2013	M	UK	4,000			All			10	10	10	472.97	247.47	0	1	1	0	1	1	0	1	1	0
Miller 1990(4)	1990	D	USA			16	All		37.0%	10	10	10	393.50	186.90	0	0	1	1	1	1	0	1	1	0
Van Eenennaam 1995(5)	1995	D	USA	71			All			10	10	10	71.93	9.90	0	0	0	0	0	0	0	0	0	0
Van Eenennaam 1995(5)	1995	D	USA	50			All		0.0%	10	10	10	79.46	4.95	0	0	0	0	0	0	0	0	0	0
Van Eenennaam 1995(5)	1995	D	USA	50			All		0.0%	10	10	10	112.33	8.87	0	0	0	0	0	0	0	0	0	0
Aghamohammadi 2018(6)	2018	D	CAN		60	145	All		19.0%	10	10	10	1,010.93	30.80	1	0	1	1	1	1	0	1	1	1
Steeneveld 2011(7)	2011	M	NTH	20000			All			10	10	10	189.15	1.78	0	1	1	0	1	1	0	0	0	0
Steeneveld 2011(7)	2011	M	NTH	20000			All		0.0%	10	10	10	208.58	1.71	0	1	1	0	1	1	0	0	0	0
Steeneveld 2011(7)	2011	M	NTH	20000			All		0.0%	10	10	10	213.64	1.70	0	1	1	0	1	1	0	0	0	0
Steeneveld 2011(7)	2011	M	NTH	20000			All		0.0%	10	10	10	219.55	1.66	0	1	1	0	1	1	0	0	0	0
Steeneveld 2011(7)	2011	M	NTH	20000			All		0.0%	10	10	10	232.22	1.55	0	1	1	0	1	1	0	0	0	0
Halasa 2012(8)	2012	M	DAN				All		34.0%	10	10	10	125.90	3.20	0	0	1	1	1	1	0	0	0	0
Halasa 2012(8)	2012	M	DAN				All		25.0%	10	10	10	147.40	4.00	0	0	1	1	1	1	0	0	0	0
Halasa 2012(8)	2012	M	DAN				All		22.0%	10	10	10	171.00	4.40	0	0	1	1	1	1	0	0	0	0
Halasa 2012(8)	2012	M	DAN				All		20.0%	10	10	10	285.70	9.90	1	0	1	1	1	1	0	0	0	0
Halasa 2012(8)	2012	M	DAN				All		17.0%	10	10	10	300.40	5.60	1	0	1	1	1	1	0	0	0	0
Halasa 2012(8)	2012	M	DAN				All		34.0%	10	10	10	98.17	2.50	0	1	1	1	1	1	0	0	0	0
Halasa 2012(8)	2012	M	DAN				All		25.0%	10	10	10	116.40	3.20	0	1	1	1	1	1	0	0	0	0
Halasa 2012(8)	2012	M	DAN				All		22.0%	10	10	10	141.30	3.60	0	1	1	1	1	1	0	0	0	0

Halasa 2012(8)	2012	M	DAN				All		20.0%	10	10	10	266.90	9.00	1	1	1	1	1	1	0	0	0	0
Halasa 2012(8)	2012	M	DAN				All		17.0%	10	10	10	271.30	4.80	1	1	1	1	1	1	0	0	0	0
Wolfova 2006(9)	2006	M	RCZ		474		All	35%	68.0%	10	10	10	87.37	4.10	0	0	0	1	1	0	0	0	0	0

Table S1: Data used for the meta-analysis (continued)

A General factors and aetiology				C- Monetary level of losses (values of each contributors)								D- Prices (of input parameters of raw models)					
Authors	Year	Study type (Modelling/Descriptive)	Country	Milk withdrawal	Milk not produced	Veterinary cost	Drug cost	Extra labour	Culling	Extended day open	Cow Mortality	Culled cow (€/kg carcass)	Replacement heifer (€/ head)	Milk (€/kg)	Feed (€/kg dry matter)	Labour (€/h)	Treatment (€/treatment, all included)
Miller 1993(1)	1993	D	USA		103.70	14.33	19.69	8.49	96.01		21.01					36.76	
Miller 1993(1)	1993	D	USA													36.76	
Miller 1993(1)	1993	D	USA													36.76	
Miller 1993(1)	1993	D	USA													36.76	
Miller 1993(1)	1993	D	USA													36.76	
Miller 1993(1)	1993	D	USA													36.76	
Miller 1993(1)	1993	D	USA													36.76	
Miller 1993(1)	1993	D	USA													36.76	
Miller 1993(1)	1993	D	USA													36.76	
Miller 1993(1)	1993	D	USA													36.76	
Miller 1993(1)	1993	D	USA													36.76	
Miller 1993(1)	1993	D	USA													36.76	
Miller 1993(1)	1993	D	USA													36.76	
Miller 1993(1)	1993	D	USA													36.76	
Miller 1993(1)	1993	D	USA													36.76	
Miller 1993(1)	1993	D	USA													36.76	
Miller 1993(1)	1993	D	USA													36.76	
Miller 1993(1)	1993	D	USA													36.76	
Miller 1993(1)	1993	D	USA													36.76	
Miller 1993(1)	1993	D	USA													36.76	
Miller 1993(1)	1993	D	USA													36.76	
Miller 1993(1)	1993	D	USA													36.76	

Miller 1993(1)	1993	D	USA													36.76	
Miller 1993(1)	1993	D	USA													36.76	
Miller 1993(1)	1993	D	USA													36.76	
Miller 1993(1)	1993	D	USA													36.76	
Miller 1993(1)	1993	D	USA													36.76	
Miller 1993(1)	1993	D	USA													36.76	
Miller 1993(1)	1993	D	USA													36.76	
Miller 1993(1)	1993	D	USA													36.76	
Miller 1993(1)	1993	D	USA													36.76	
Miller 1993(1)	1993	D	USA													36.76	
Miller 1993(1)	1993	D	USA													36.76	
Miller 1993(1)	1993	D	USA													36.76	
Miller 1993(1)	1993	D	USA													36.76	
Liang 2017(2)	2017	M	USA	47.00	142.33	16.82	50.43	10.12	9.00	-1.35	10.58	1.69	1,502	0.328-0.3682	0.16	28.88 – 30.36	
Liang 2017(2)	2017	M	USA									1.69	1,502	0.412-0.194	0.16	28.88 – 30.36	
Liang 2017(2)	2017	M	USA									1.69	1,502	0.328-0.3682	0.18	28.88 – 30.36	
Liang 2017(2)	2017	M	USA									1.69	1,502	0.328-0.3682	0.16	28.88 – 30.36	
Liang 2017(2)	2017	M	USA									1.94	1,502	0.328-0.3682	0.16	28.88 – 30.36	
Liang 2017(2)	2017	M	USA	57.43	144.96	16.82	50.43	10.12	9.06	75.62	11.25	1.69	1,502	0.328-0.3682	0.16	28.88 – 30.36	
Liang 2017(2)	2017	M	USA									1.69	1,502	0.412-0.194	0.16	28.88 – 30.36	
Liang 2017(2)	2017	M	USA									1.69	1,502	0.328-0.3682	0.18	28.88 – 30.36	
Liang 2017(2)	2017	M	USA									1.69	1,502	0.328-0.3682	0.16	28.88 – 30.36	
Liang 2017(2)	2017	M	USA									1.94	1,502	0.328-0.3682	0.16	28.88 – 30.36	
Liang 2017(2)	2017	M	USA									1.69	1,502	0.328-0.3682	0.16	28.88 – 30.36	
Liang 2017(2)	2017	M	USA									1.69	1,502	0.328-0.3682	0.16	28.88 – 30.36	
Liang 2017(2)	2017	M	USA									1.69	1,502	0.328-0.3682	0.16	28.88 – 30.36	
Liang 2017(2)	2017	M	USA									1.69	1,502	0.328-0.3682	0.16	28.88 – 30.36	
Down 2013(3)	2013	M	UK	67.57	126.99		11.40	11.40	65.12					0.309		5.89 – 10.58	7.73
Down 2013(3)	2013	M	UK											0.309		5.89 – 10.58	7.73
Down 2013(3)	2013	M	UK											0.309		5.89 – 10.58	7.73
Down 2013(3)	2013	M	UK	83.85	126.99		18.72	14.65	65.12					0.309		5.89 – 10.58	12.86
Down 2013(3)	2013	M	UK	63.50	131.88		48.03	9.77	63.50					0.309		5.89 – 10.58	46.08
Down 2013(3)	2013	M	UK	67.57	131.88		60.24	10.58	64.31					0.309		5.89 – 10.58	54.38
Down 2013(3)	2013	M	UK	77.34	134.32		52.10	13.84	65.94					0.309		5.89 – 10.58	46.89

Down 2013(3)	2013	M	UK											0.309		5.89 – 10.58	7.73
Down 2013(3)	2013	M	UK											0.309		5.89 – 10.58	12.86
Down 2013(3)	2013	M	UK											0.309		5.89 – 10.58	46.08
Down 2013(3)	2013	M	UK											0.309		5.89 – 10.58	54.38
Down 2013(3)	2013	M	UK											0.309		5.89 – 10.58	46.89
Miller 1990(4)	1990	D	USA		49.32	27.59		4.04	61.92								
Van Eenennaam 1995(5)	1995	D	USA											0.412-0.194			2.06
Van Eenennaam 1995(5)	1995	D	USA											0.412-0.194			6.66
Van Eenennaam 1995(5)	1995	D	USA											0.412-0.194			4.89
Aghamohammadi 2018(6)	2018	D	CAN	76.38	307.94	12.46	18.22	3.64	510.20					0.328-0.3682		19.42 – 30.36	18.00
Steeneveld 2011(7)	2011	M	NTH	26.18	80.22		23.64	14.36	44.76							19.42 – 30.36	16.04
Steeneveld 2011(7)	2011	M	NTH	36.31	76.84		34.62	20.27	40.53							19.42 – 30.36	27.02
Steeneveld 2011(7)	2011	M	NTH	26.18	76.00		56.58	15.20	39.69							19.42 – 30.36	48.13
Steeneveld 2011(7)	2011	M	NTH	26.18	75.15		65.87	15.20	37.16							19.42 – 30.36	58.27
Steeneveld 2011(7)	2011	M	NTH	36.31	73.47		65.87	21.11	35.47							19.42 – 30.36	59.11
Halasa 2012(8)	2012	M	DAN		28.75	8.07	16.15	14.86	57.81							19.42 – 30.36	16.47
Halasa 2012(8)	2012	M	DAN		32.07	8.35	27.23	20.21	14.82							19.42 – 30.36	27.45
Halasa 2012(8)	2012	M	DAN		30.95	7.99	53.91	20.96	57.40							19.42 – 30.36	54.91
Halasa 2012(8)	2012	M	DAN		25.26	24.71	16.47	14.28	210.84							19.42 – 30.36	16.47
Halasa 2012(8)	2012	M	DAN		30.36	32.30	27.78	20.67	182.16							19.42 – 30.36	27.45
Halasa 2012(8)	2012	M	DAN		28.75	8.07	16.15	14.86	57.81							19.42 – 30.36	16.47
Halasa 2012(8)	2012	M	DAN		32.07	8.35	27.23	20.21	59.30							19.42 – 30.36	27.45
Halasa 2012(8)	2012	M	DAN		30.95	7.99	53.91	20.96	57.40							19.42 – 30.36	54.91
Halasa 2012(8)	2012	M	DAN		25.26	24.71	16.47	14.28	210.84							19.42 – 30.36	16.47
Halasa 2012(8)	2012	M	DAN		30.36	32.30	27.78	20.67	182.16							19.42 – 30.36	27.45
Wolfova 2006(9)	2006	M	RCZ	72.14		27.33		8.15						0.328-0.368		5.89 – 10.58	13.46

Section B: Moderators “drug cost” and “milk withdrawal” not included in Table S1 since considered for all the 82 observations.

Table S2: Full references of the considered studies in the final meta-regression analysis.

1. Miller GY, Bartlett PC, Lance SE, Anderson J, Heider LE. Costs of clinical mastitis and mastitis prevention in dairy herds. *Journal of the American Veterinary Medical Association* (1993) **202**:1230–1236.

2. Liang D, Arnold LM, Stowe CJ, Harmon RJ, Bewley JM. Estimating US dairy clinical disease costs with a stochastic simulation model. *Journal of Dairy Science* (2017) **100**:1472–1486. doi:10.3168/jds.2016-11565

3. Down PM, Green MJ, Hudson CD. Rate of transmission: A major determinant of the cost of clinical mastitis. *Journal of Dairy Science* (2013) **96**:6301–6314. doi:10.3168/jds.2012-6470

4. Miller GY, Dorn CR. Costs of dairy cattle diseases to producers in Ohio. *Preventive Veterinary Medicine* (1990) **8**:171–182. doi:10.1016/0167-5877(90)90009-7

5. Van Eenennaam AL, Gardner IA, Holmes J, Perani L, Anderson RJ, Cullor JS, Guterbock WM. Financial Analysis of Alternative Treatments for Clinical Mastitis Associated with Environmental Pathogens. *Journal of Dairy Science* (1995) **78**:2086–2095. doi:10.3168/jds.S0022-0302(95)76835-7

6. Aghamohammadi M, Haine D, Kelton DF, Barkema HW, Hogeveen H, Keefe GP, Dufour S. Herd-Level Mastitis-Associated Costs on Canadian Dairy Farms. *Front Vet Sci* (2018) **5**:100. doi:10.3389/fvets.2018.00100

7. Steeneveld W, van Werven T, Barkema HW, Hogeveen H. Cow-specific treatment of clinical mastitis: An economic approach. *Journal of Dairy Science* (2011) **94**:174–188. doi:10.3168/jds.2010-3367

8. Halasa T. Bioeconomic modeling of intervention against clinical mastitis caused by contagious pathogens. *Journal of Dairy Science* (2012) **95**:5740–5749. doi:10.3168/jds.2012-5470

9. Wolfová M, Štípková M, Wolf J. Incidence and economics of clinical mastitis in five Holstein herds in the Czech Republic. *Preventive Veterinary Medicine* (2006) **77**:48–64. doi:10.1016/j.prevetmed.2006.06.002
